# Reduced Effective Postural Responses to External Perturbations in Chronic Ankle Instability

**DOI:** 10.1111/sms.70345

**Published:** 2026-07-10

**Authors:** Xiaohan Xu, Joanna Bowtell, William R. Young, Daniel T. P. Fong, Genevieve K. R. Williams

**Affiliations:** ^1^ Public Health and Sports Sciences Department University of Exeter Exeter UK; ^2^ National Centre for Sport and Exercise Medicine, School of Sport, Exercise and Health Sciences Loughborough University Loughborough UK

**Keywords:** chronic ankle instability, joint torques, margin of stability, perturbation, postural control

## Abstract

Chronic ankle instability (CAI) is associated with postural control deficits due to altered postural strategy, particularly during transitions between postures (e.g., landing followed by immediate medial jump) and in response to sudden external perturbations. However, postural stability and the underpinning joint action remain unclear. This study aimed to identify differences in postural response to a mediolateral moving platform during single‐leg stance between individuals with CAI and healthy controls (HC). Twenty‐three CAI and 23 HC participants performed balance tasks during 1‐s mediolateral perturbation. Ground reaction force and kinematic data were recorded, and margin of stability (MoS), and joint torques in the frontal plane were calculated for their first successful trial. The CAI group showed increased MoS during sudden changes in platform movement direction (*p* = 0.045) and the phase after platform stop (*p* = 0.033) compared to the HC, suggesting a less effective postural response to external perturbations. These findings suggest a less stable postural strategy in people with CAI during single‐leg stance with perturbation. The weaker relationship between MoS and hip‐ and trunk‐ torque in the CAI than HC group in response to the perturbation provided evidence for reduced effectiveness of joint actions in postural control. Sudden base of support changes, such as accelerations and decelerations, are shown to be effective in identifying postural impairments in CAI. Further research is needed to determine whether these impairments can be improved through practice.

## 1 Introduction

Lateral ankle sprains are among the most common musculoskeletal injuries [1], with incidence rates of approximately 2–20 per 1000 person‐years in general populations, with higher rates in physically active and athletic groups [2]. These injuries damage the lateral ligaments responsible for joint stability, leading to pathological laxity, tissue reinjury and adaptation that alter sensory‐perceptual and motor‐behavioral function [3], with approximately 50% of cases progressing to repeated ankle sprain and chronic ankle instability (CAI) [4]. CAI is associated with decreased physical activity [5], reduced quality of life [6], and increased risks of early‐onset ankle osteoarthritis [7]. End stage ankle arthritis is also associated with substantial healthcare costs [8]. Although the optimal treatment for CAI remains unclear, a better understanding of the underlying sensorimotor mechanisms and potential biomarkers is essential for improving management strategies.

Postural control deficits are common in people with CAI. Evidence suggests that these deficits stem from diminished somatosensation [9] and impaired ankle neuromuscular control [10]. Studies on unilateral weight‐bearing tasks, such as single‐leg stance [11], landing [12], and lateral stepping down [13, 14], have revealed significant changes in postural control and lower limb kinematics in individuals with CAI. To compensate for partially deafferented ankle joints, individuals with CAI often shift from ankle‐ to hip‐based strategies in the sagittal plane to maintain postural stability [15]. Additionally, individuals with CAI exhibited increased trunk range of motion and angular velocity in the frontal plane during single‐leg stance on a mediolateral moving platform [16]. However, the underlying mechanisms of these kinematic alterations remain unclear. For example, it is uncertain whether increased trunk movement serves as a compensatory strategy to increase angular momentum for controlling the center of mass (CoM) [17] or reflects an impaired ability to stabilize the trunk effectively.

In the inverted pendulum model of human standing balance, stability is maintained by keeping the vertical projection of the CoM within the base of support (BoS). Any offset between the CoM and center of pressure (CoP) will create an accelerating force that moves the CoM further away [18]. While ankle muscles can adjust the CoP toward the BoS margins, during single‐leg balance, the reduced mediolateral BoS limits their effectiveness. Excessive ankle tilting will saturate the ankle torque, necessitating the rapid engagement of hip torque to dynamically control the CoM [19]. During dynamic balance, the restoration of postural stability requires accounting for CoM velocity, with the margin of stability (MoS) quantifying the absolute distance between the extrapolated CoM and the edges of the BoS to evaluate instantaneous mechanical stability [20, 21]. MoS characteristics are task‐dependent and reflect the movement patterns of different pathologies. A greater MoS indicates a less conservative and more unstable balance strategy. This pattern has been observed in pathological conditions, such as stroke [22, 23], amputation [24], and multiple sclerosis [25]. Increased MoS is associated with instability during physically demanding tasks, including dual‐task walking [26], perturbation‐induced gait [27], and walking with higher stride frequencies [28]. In contrast, a decreased MoS indicates a more vigilant, conservative strategy that enhances stability. This pattern has been reported in pathological gait, including Parkinson's disease [29, 30] and in spinal cord injury during slow walking [31].

Joint torques have been used to quantify the postural strategy in the sagittal plane [32, 33, 34]. In the frontal plane, when the CoM shifts laterally away from the CoP, ankle invertor torque can adjust the CoP laterally and the CoM medially to restore balance. However, excessive ankle inversion torque increases the risk of ankle sprain [35]. Studies have reported that people with CAI exhibited greater ankle inversion torque during fast postural transitions, such as landing followed by immediate medial jump [36], and during transition from terminal stance to pre‐swing in walking [37], compared to healthy controls. Conversely, Ono, Yoshida, Ota, Tanigawa [38] reported reduced ankle inversion torque during the contact phase of a medial hop. In an attempt to control and provide a consistent and constrained disturbance, this study uses precise, repeatable external perturbations via a moving platform. By examining the selection of the strategies used in response to external perturbations, research can show evidence for whether CAI populations have different patterns in generating joint torques for effective stabilization [39, 40], without capturing the inter and intra‐individual variation inherent in internally generated movements such as hops.

Recent studies have investigated age‐related differences in joint contributions to postural stability by examining the temporal coupling of joint motion or torque with MoS [34, 41]. Older adults exhibited a stronger relationship between sagittal plane hip torque and MoS during gait, suggesting a greater reliance on hip control for postural stability [34]. Our previous study examined joint kinematics contributing to balance performance by correlating discrete root mean square angular velocity with time‐to‐boundary (TTB) [16]. However, discrete measures lack insight into the temporal variation involved in continuous movement modulation. Analyzing joint torque as a time series enables assessment of the temporal dynamics of joint actions underlying postural adjustments. Joint torques directly capture the forces driving movement, whether from muscle activation, external forces, or interactive torques from other body segments [32], which provide a clearer understanding of how the nervous system regulates postural stability through joint control. Building on this, the present study sought to determine the role of joint torques in maintaining stability over time.

The primary aim of this study was to examine differences in instantaneous mechanical stability, quantified by MoS, between individuals with CAI and healthy controls during an ML perturbation in single leg stance. We hypothesized that CAI would exhibit an increased MoS in response to platform motion, consistent with the greater trunk movement and velocity previously observed [16]. The secondary aim was to investigate group differences in the relationship between MoS and normalized joint torques at the ankle, hip, and trunk. Since MoS reflects instantaneous mechanical stability, incorporating CoM position, velocity, and BoS, its temporal coupling with ankle, hip, and trunk torques indicates how effectively joint actions generate optimal stabilization with minimal energy expenditure in response to BoS movements elicited by moving platform [42]. Building on previous findings demonstrating strategy shifts from ankle to hip in sagittal plane during landing and jumping [15], and potential compensatory adaptations reflected by distinct trunk and hip movement patterns during single‐leg stance on a moving platform [16], we hypothesized that CAI participants would show a stronger cross‐correlation between MoS and hip‐ and trunk‐torques, and a weaker cross‐correlation between MoS and ankle torque in frontal plane compared to healthy controls.

## 2 Methods

### 2.1 Participants

Twenty‐three participants with CAI and 23 healthy controls (HC) participated in the study after providing written informed consent. A priori sample size estimation for 1‐dimensional waveform MoS data was performed using Power1D (v.0.1.7) [43] in Python 3.12.7. Based on pilot data from 10 HC participants and a simulated alternative hypothesis with a Gaussian pulse and smooth‐Gaussian modeled noise with the same characters as the original data (i.e., mean, standard deviations and full‐width‐at‐half‐maximum) [44], the minimum sample size is 23 participants per group for a two‐tailed two‐sample *t*‐test at 80% power with a center‐of‐interest (COI) of three. Participants were instructed about the study purpose and procedures and signed written informed consent prior to participating. The Sports and Health Sciences Department Ethics Committee approved the study before commencement (1071581). All methods were performed in accordance with the guidelines and regulations.

The participant characteristics were similar between groups: sex, age, height, body mass, and physical activity level, assessed using the International Physical Activity Questionnaire‐Short Form (IPAQ‐SF) (Table 1). Participants in the CAI group were selected based on the International Ankle Consortium criteria [45], with impaired ankle function identified by a Cumberland Ankle Instability Tool score of < 24 [46]. The control group (HC) had no history of lateral ankle sprains and were selected to match the CAI cohort in sex and physical activity at a proportional level, with height and body mass within one standard deviation of the CAI mean.

**TABLE 1:** Participants' demographics and anthropometrics.

	CAI (*n* = 23)	HC (*n* = 23)	*p*
Sex	Female: 10	Female: 9	0.765
	Male: 13	Male: 14	
Age, years—mean ± SD	22.9 ± 3.2	24.5 ± 3.9	0.159
Height, m—mean ± SD	1.71 ± 0.09	1.72 ± 0.08	0.663
Body Mass, kg—mean ± SD	70.4 ± 9.1	65.2 ± 8.5	0.052
IPAQ‐SF	Moderate: 13	Moderate: 12	1.000
	High: 10	High: 11	
CAIT Score	17.5 ± 4.1	30.0 ± 0	< 0.0^a^

*Note:* Chi‐square tests for sex and IPAQ‐SF; Independent *t*‐tests for age, height, body mass and CAIT.

Abbreviations: CAIT, Cumberland Ankle Instability Tool; IPAQ‐SF, International Physical Activity Questionnaire‐Short Form; SD, standard deviation.

^a^ Denotes significant difference between CAI and the HC (*p* < 0.05).

All participants were aged between 18 and 35 years, with no acute musculoskeletal injuries within the 3 months prior to testing, and were free from visual or hearing impairments, dizziness, recurrent falls, vestibular dysfunction, lower extremity fractures, pain, surgery, or prior experience in professional balance training.

### 2.2 Data Collection and Analysis

**FIGURE 1:**
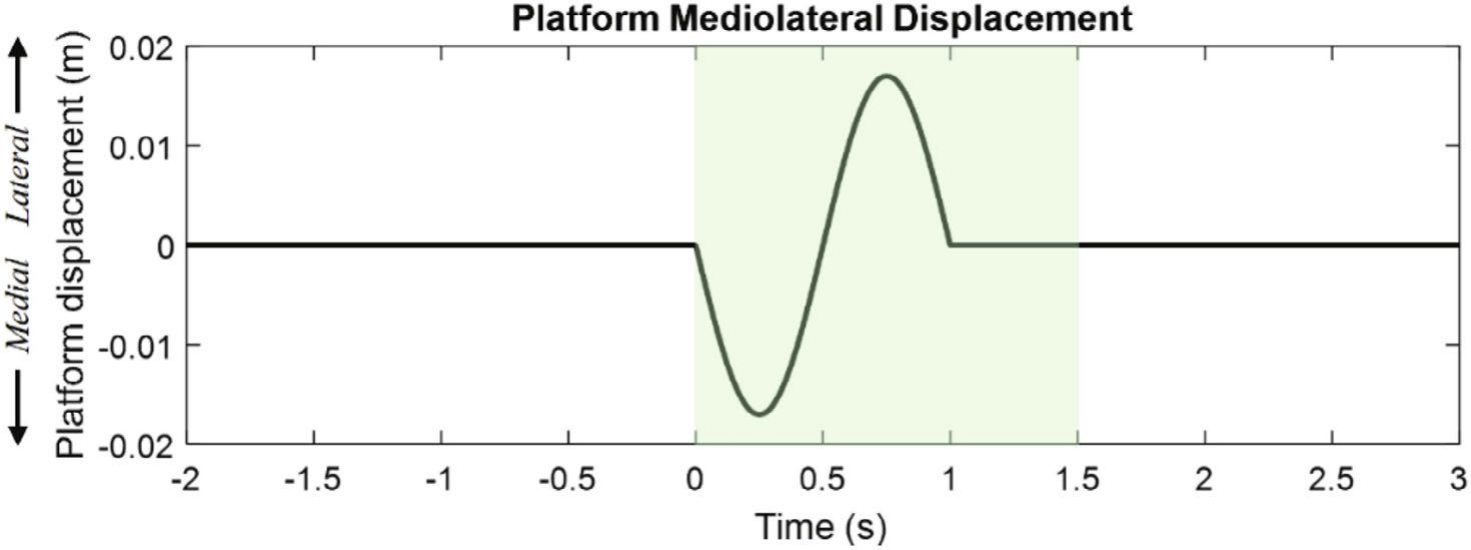
Platform motion during a balance trial (−2 s to +3 s) included a 2‐s baseline phase, followed by the onset of 1‐s mediolateral sinusoidal platform movement at 0 s with a peak‐to‐peak displacement of 34 mm, concluding with a 2‐s recovery phase. The shaded region (0–1.5 s) represents the period for data analysis.

**FIGURE 2:**
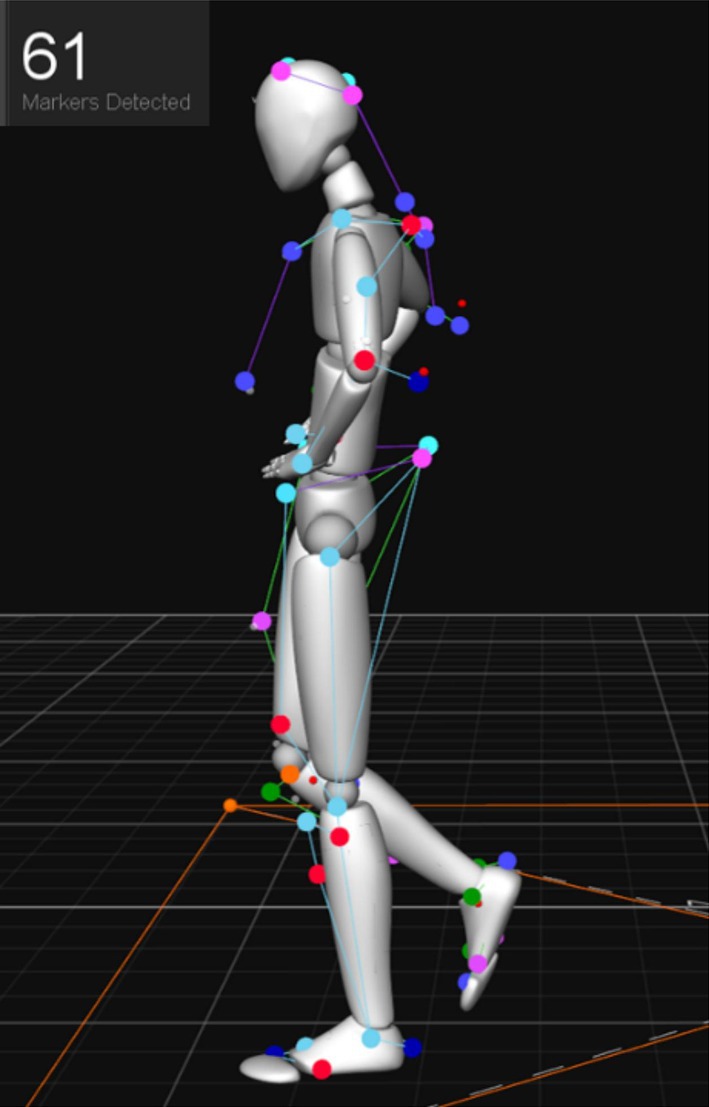
Standardized single‐leg standing visualized through 61 markers (57 Biomech‐57 marker set and four markers on platform) by motion capture and Motive Software (version 2.1.1; https://optitrack.com/software/motive/).

A subset of these data, MoS during the 0–1 s window, was previously reported [47], as part of a study on muscle activation and practice adaptation. Participants performed barefoot single‐leg balance tests on the affected side in the CAI group, adopting the standardized standing position described in the previous study [16]. In cases of bilateral CAI, the side with the lower CAIT score was tested. Height‐, gender‐, and IPAQ‐SF‐matched HCs were tested on the corresponding side. Although leg dominance could influence balance strategies, the effect was considered negligible [48]. Each balance trial lasted 5 s and included three phases: a 2‐s baseline phase (pre‐perturbation), a 1‐s perturbation phase, and a 2‐s recovery phase (post‐perturbation) (Figure 1). The perturbation parameters were determined through piloting to create appropriately challenging yet safe and precisely reproducible balance perturbation. The motion was produced in the mediolateral direction designed to elicit ankle inversion/eversion responses relevant to CAI, with a sinusoidal motion of the platform to challenge balance based on two changes in direction of the platform (Figure S1). Piloting determined that a 1.7‐cm amplitude (approach maximum platform displacement, 17% of the base of support in ML, assuming an average foot width of 10‐cm) with 1‐Hz oscillation produced sufficient CoM displacement to challenge postural stability and elicit whole‐body corrective responses, while still enabling participants to achieve their first successful balance trial within a few repetitions, minimizing familiarization effects. Participants stood facing the anteroposterior axis of the global coordinate system, ensuring their standing foot was pointed straight ahead (Figure 2). They were instructed to maintain their gaze on a target positioned at eye level, approximately three meters away on a wall. A failed trial was defined as losing balance during the 5‐s trial, taking additional steps, jumping, or creating frictional movements against the platform surface with the heel or toes, as well as visually apparent movements, such as lifted legs and arms beyond minor adjustments. In case of failure, the task was repeated until a successful trial was achieved. The researcher stood close to the participants throughout the task to provide immediate physical support in the event of a potential loss of balance.

Participants performed the balance task on a 3.6 m × 3.6 m instrumented moveable floor within the Exeter VSimulators facility [32]. Three‐dimensional positional data were recorded using a 24‐camera motion capture system (OptiTrack, NaturalPoint Inc., Corvallis, OR, USA) at a sampling frequency of 100 Hz, with 57 reflective markers placed according to the Biomech‐57 marker set [49]. Four extra markers were attached to the floor to track platform movement. Ground reaction forces and moments were recorded using the AMTI force plates (120 cm × 120 cm, Advanced Mechanical Technology Inc.) at a sampling rate of 1 kHz. Marker trajectories and force plate data were filtered using a fourth‐order Butterworth low‐pass filter with a 10 Hz cutoff. Force plate data were down sampled to 100 Hz using a spline interpolation method to align with the marker data.

Force plate inertial components were removed from force data using the method by Preuss, Fung [50]. The base of support (BoS) was defined as the instantaneous CoP position [20, 51]. The MoS was therefore calculated as signed CoP—XCoM distance. Under the adopted sign convention, a greater distance indicated reduced instantaneous mechanical stability during the perturbation. The CoM was calculated using a 14‐segment model (head, trunk, upper arms, forearms, hands, thighs, shanks, and feet), with each segment's CoM estimated from the 3D marker locations [52]. The CoM velocity was determined as the first derivative of the CoM position. CoM and CoP data in the frontal plane were included in the following analysis. Joint torques, normalized to body mass, were calculated using 3D inverse dynamics [53]. The method of modeling was based on the Newton‐Euler approach, incorporating body segment inertial properties, kinematics, and external forces into the equations of motion to determine joint reaction forces at the center of rotation and net joint torques for the ankle, knee and hip [53]. Trunk torque was calculated as the sum of the moments acting on the trunk segment (from the greater trochanter to the glenohumeral joint), with the glenohumeral joint on the same side as the stance leg serving as the axis of rotation. Ankle inversion/eversion torques, hip abduction/adduction torques, and trunk lateral/medial flexion torques were computed for analysis. The MoS was calculated in the frontal planes for each trial with the formula [20]:

MoS=CoP−XCoM

The XCoM is extrapolated CoM, calculated as follows:

$$\text{XCoM}=x+\left(\frac{{\mathrm{CoM}}_{v}}{\omega \theta }\right)$$

$$\omega \theta =\sqrt{\frac{g}{l}}$$

where x is the mediolateral (ML) position of the vertical projection of the CoM (m) on the ground, ${\mathrm{CoM}}_{v}$ is CoM velocity (m/s) in the frontal plane, ωθ is the eigen frequency of the inverted pendulum, g is the gravitational constant = 9.81 m/s [2], and l is the pendulum length (m), estimated as 1.34 times height from lateral malleolus to greater trochanter in the frontal plane [20]. The CoP‐to‐ankle center distance in the frontal plane was calculated. All the data were processed in MATLAB 2022b (MathWorks Inc., Natick, Massachusetts).

### 2.3 Statistical Analysis

Independent *t*‐tests were performed by using SPM1D package (v.0.4.11, available at https://spm1d.org) in MATLAB, to test for differences in MoS, ankle, hip and trunk torque in the frontal plane between CAI and HC groups during the period from perturbation onset to 1.5 s post‐perturbation (Figure 1). Cross‐correlation was used to quantify the relationship between MOS and joint torque waveforms at zero‐phase lag [34], across three time‐windows (0–0.5 s, 0.5 s–1 s and 1–1.5 s), selected based on platform movement phases. The first two windows represent half‐cycles of platform motion, with transitions occurring at the center of each phase, while the final window captures the postural response after the platform stops, ensuring equal duration across all phases (Figure 1). For each participant, the absolute values of cross‐correlation at zero‐lag were calculated, with strength categorized as strong (0.5–1), moderate (0.3–0.5), or weak (0–0.3). Independent *t*‐tests were conducted to evaluate between‐group differences in the strength of cross‐correlation for MoS and joint torque pairings across three time‐windows, once the normal distribution of data was confirmed by using the Shapiro–Wilk test. Cohen's d effect size was calculated to validate the *t*‐test results, with interpretation as small (< 0.40), moderate (0.40–0.80), and large (> 0.80) [54]. Data for the first successful trial were taken into analysis. Statistical significance was set at *p* < 0.05.

## 3 Results

### 3.1 Number of Attempts

There was no significant difference in the number of attempts required to achieve the first successful trial between groups (CAI 3.6 ± 2.4, HC 2.8 ± 1.1; Z = −0.812, *p* = 0.417, U = 229, Mann–Whitney *U* test).

### 3.2 Margin of Stability

**FIGURE 3:**
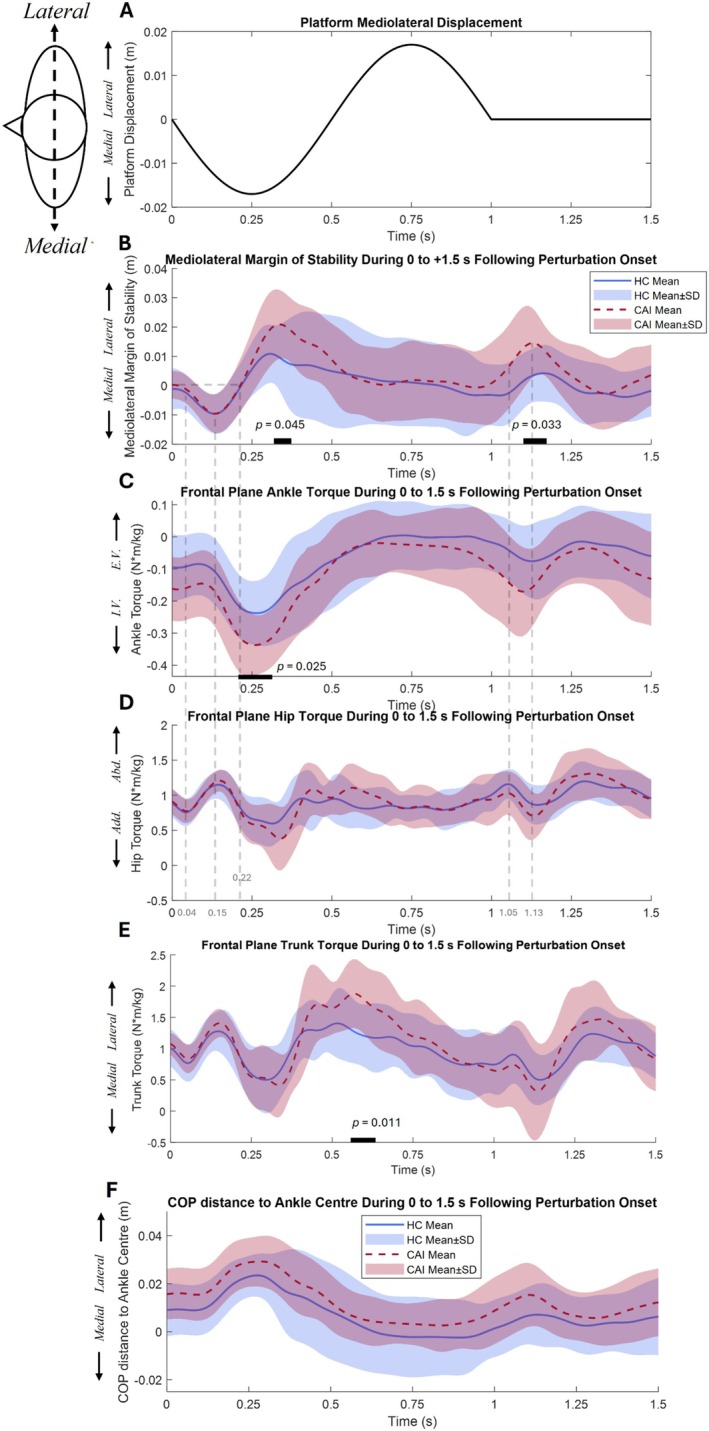
(A) Platform mediolateral displacement from 0 to 1.5 s, including 1‐s perturbation phase followed by 0.5‐s recovery phase. (B) Mean and standard deviation of mediolateral Margin of Stability (MoS) during 0–1.5 s for HC (blue), and CAI (red) individuals. Statistical differences between groups throughout 1.5 s are indicated along the x‐axis with the black bar. (C) The ankle torque in frontal plane during 0–1.5 s for HC and CAI individuals. (D) The hip torque in frontal plane during 0–1.5 s for HC and CAI individuals. Five key time points are highlighted: 0.04 s (onset of hip abduction torque increase), 0.15 s (largest medial MoS), 0.22 s (MoS = 0), 1.05 s (hip adduction torque increase), and 1.13 s (hip returns to neutral, abduction torque increase). (E) The Trunk torque in frontal plane during 0–1.5 s for HC and CAI individuals. Lateral Lean in positive. (F) Distance between the Centre of Pressure (CoP) and the ankle center of the stance leg during 0–1.5 s for HC and CAI groups.

The MoS responded to the platform motion as shown in Figure 3B. When MoS > 0, the BoS was positioned laterally to the XCoM, and when MoS < 0, the BoS was positioned medially to the XCoM. As the platform started moving medially, the BoS shifted medially, moving further away from the XCoM. At 0.15 s, the XCoM began adjusting and moved closer to the BoS. At 0.25 s, the platform reversed direction, shifting the BoS laterally while the XCoM continued to move medially as the BoS moved laterally further away from the XCoM. After a brief delay (< 0.1 s) following the platform transition, the XCoM started moving laterally, reducing its distance from the BoS. Between 0.5 and 1 s, individual postural adjustments showed variability, with no consistent pattern observed during the second platform transition at 0.75 s. When the platform motion stopped at 1 s, the BoS stopped while the XCoM moved medially, increasing the distance between the XCoM and the BoS. The CAI group exhibited a larger MoS in response to the BoS transition from medial to lateral (t* _(1,44)_ = 3.216, *p* = 0.045) and during the BoS moving medially followed by a sudden stop (t* _(1,44)_ = 3.216, *p* = 0.033).

### 3.3 Joint Torques

The ankle, (Figure 3C) hip, (Figure 3D) and trunk torque (Figure 3E) responded to platform motion. As the platform moved medially, ankle inversion torque increased to prevent body collapse by shifting the CoM medially. The torque peaked during the transition of platform motion from medial to lateral, at which point the CAI group exhibited significantly greater inversion directed torque magnitude compared to the HC group (t* _(1,44)_ = 3.112, *p* = 0.025). Hip adduction torque initially increased in response to the medial stretching effect of BoS (0–0.04 s), followed by an increase in abduction torque (0.04–0.15 s) to bring the hip back to a neutral position. This response mechanically moves the XCoM further away from the BoS, after which an increase in adduction torque shifted the XCoM medially, closer to the BoS (0.15–0.22 s). When the platform stopped moving medially, the sudden deceleration caused the hip to move into abduction (1–1.05 s), followed by an increase in adduction torque (1.05–1.13 s) to return the hip to neutral, causing the XCoM to move further away from the BoS. Subsequently, abduction torque increased to bring the XCoM closer to the BoS. No significant differences in hip torque were observed between the CAI and HC groups during balance trial. The time‐series profile of trunk torque was similar to that of hip torque. However, the CAI group exhibited significantly greater trunk lateral lean torque during 0.55–0.61 s compared to the HC group (t* _(1,44)_ = 3.449, *p* = 0.011) as the BoS shifted laterally, indicating the need for increased lateral lean torque to bring the CoM closer to the BoS.

### 3.4 Cross‐Correlation Between MoS and Joint Torques

Cross‐correlation analysis demonstrated a moderate to strong relationship between MoS and joint torques (trunk, hip, or ankle) in both groups (Table 2). However, the CAI group showed a significantly weaker cross‐correlation between MoS and both hip and trunk torques compared to the HC group during 0–0.5 s (hip: *p* = 0.044; trunk: *p* = 0.017) and 1–1.5 s (hip: *p* = 0.050; trunk: *p* = 0.041) following perturbation. There were no differences in MoS‐Ankle Pairing between groups.

**TABLE 2:** Average and standard deviation (SD) for cross‐correlation between joint torques and the margin of stability in frontal plane for CAI and HC.

	Time window	CAI pairing R			HC pairing R			*p*	Cohen's *d*
		Mean ± SD			Mean ± SD				
MoS‐Ankle Pairing	0–0.5s	0.486	±	0.231	0.571	±	0.281	0.270	−0.392
MoS‐Hip Pairing		0.308^a^	±	0.210	0.457	±	0.273	0.044	−0.610
MoS‐Trunk Pairing		0.285^a^	±	0.197	0.468	±	0.295	0.017	−0.730
MoS‐Ankle Pairing	0.5–1s	0.592	±	0.279	0.602	±	0.315	0.913	−0.032
MoS‐Hip Pairing		0.628	±	0.201	0.571	±	0.325	0.484	0.209
MoS‐Trunk Pairing		0.608	±	0.236	0.557	±	0.297	0.524	0.189
MoS‐Ankle Pairing	1–1.5s	0.628	±	0.271	0.606	±	0.299	0.795	0.077
MoS‐Hip Pairing		0.488^a^	±	0.292	0.664	±	0.299	0.050	−0.595
MoS‐Trunk Pairing		0.491^a^	±	0.200	0.644	±	0.283	0.041	−0.622

*Note:* Effect size was estimated by Cohen's *d*. MoS‐Joint Paring: absolute values of cross‐correlation at zero‐lag between Margin of Stability and joint torques.

^a^ Denotes significant difference between CAI and the HC (*p* < 0.05).

## 4 Discussion

This study investigated differences in postural stability and the contributions of ankle, hip, and trunk torques to postural control during a perturbation balance task in individuals with CAI and healthy controls. Consistent with our hypothesis, the CAI group demonstrated an increased mediolateral MoS in response to BoS changes, indicating a less stable balance strategy compared to the HC group. However, contrary to our hypothesis, the CAI group showed a weaker relationship between MoS and hip or trunk torque during 0–0.5 s and 1–1.5 s, suggesting a reduced hip and trunk temporal coupling with MoS. Although no significant differences were observed between groups in the relationship of ankle torque to MoS, the CAI group exhibited greater ankle inversion torque than the HC group during the platform transition from medial to lateral.

In the current study, MoS‐ML reflects how the CoM's distance and velocity correspond to the BoS movement during single‐leg stance on a moving platform. The CAI group demonstrated a delayed lateral XCoM transition, and a correspondingly greater MoS‐ML compared to the HC group. Increased MoS‐ML compared to controls has been observed in other groups with lower limb amputation [24], multiple sclerosis [25], and post‐stroke [22, 23] during gait, or during balance destabilizing tasks, such as dual‐task walking [26], and perturbated gait [27]. The increased MoS‐ML in the CAI population provides evidence of decreased instantaneous mechanical stability during this task which challenges posture. Previous studies have reported dynamic postural instability in individuals with CAI during the star excursion balance test, evidenced by increased CoP velocity [55], greater path length [56], and reduced fractal dimension [57]. Additionally, individuals with CAI require longer time to stabilization (TTS) following landing tasks [12], indicating less effective balance recovery. Together with observed greater MoS in the CAI group during BoS transitions or stops, the evidence highlights deficits in dynamic postural control, particularly in response to sudden motion. Further research is needed to determine how the behavioral deficits observed in the present study relate to underlying somatosensory [58, 59, 60] and reflexive functions [61].

The two phases of the perturbation motion where MoS was significantly different between CAI and HCs occurred when the platform transitioned from medial to lateral motion or a sudden stop from medial movement. These phases require precise control of lateral CoM movement, primarily relying on ankle evertor, hip abductor, and trunk lateral lean torques. This greater MoS observed in CAI group might be attributed to the different mediolateral hip and trunk torque strategy, as indicated by the weaker relationship between MoS and hip‐ and trunk‐ torque compared to the HC group. Previous research has shown that individuals with ankle sprains often shift their movement patterns from an ankle‐based to a hip‐based strategy in the sagittal plane [62, 63]. Alterations in hip kinematics in the sagittal plane are widely reported in individuals with CAI [64]. Also, they adopted a hip‐dominant strategy with increased hip extension moment and power during landing and jumping [15]. Rapid changes in hip torque dynamically controls the CoM during single‐leg stance by altering body angular momentum through such as trunk or arm movements [17], with the hip strategy being the most effective in generating larger instantaneous shear forces that influence CoM movement [65]. Given this biomechanical basis, our findings suggest that the CAI group may use less effective hip and trunk actions compared to the HCs. The CAI group had a significantly larger MoS, indicating greater instability and less effective hip and trunk responses to the perturbation, followed by higher trunk torque, as observed between 0.55–0.61 s in the trial, potentially, reflecting compensation for delayed or untimely postural adjustments. This less effective adjustment may supply a potential explanation for the kinematic alterations in the hip and trunk reported in the literature [16].

The CAI group exhibited significantly increased inversion torque compared to the HC group, but this increase did not contribute to greater dynamic stability. In fact, during the medial‐to‐lateral platform transition (from 0.25 s), eversion torque should act to shift CoM laterally. The HC group achieved peak MoS earlier (~0.30 s) than the CAI group (~0.37 s), indicating delayed lateral CoM control in CAI, likely due to less effective generation of eversion torque. Previous kinematics studies have reported increased inversion angles in CAI during walking [66], landing [12], and side‐cutting [67]. Higher inversion torque has been observed during sidestep cutting [35], and during fast postural transitions, landing with immediate transition to medial side jump [36], and during walking between terminal stance and pre‐swing in CAI compared to healthy controls [37]. While sufficient inversion torque is necessary to prevent body lateral collapse, excessive torque can tilt the foot onto the edge of the BoS, thereby saturating the effective ankle torque and stabilizing capacity [19]. The HC group maintained balance successfully with lower invertor torque, indicating the increased invertor torque in the CAI group might be dysfunctional. The greater inversion torque observed in CAI may be attributed to their tendency to place the CoP in a more laterally deviated position during single‐leg standing (Figure 3F). The standard deviation of CoP placement in the CAI group was reduced, which might reflect that CAI has a reduced functional BoS (i.e., extreme boundaries of CoP allowing the weight shifting as much as possible) [21] during single‐leg standing, particularly on the lateral side of the foot. If so, this could inform footwear designs to enhance lateral ankle support.

Regarding study limitations, trunk torque was estimated using a single rigid segment model from the greater trochanter to the glenohumeral joint [16, 68, 69], although the trunk has multiple segments, this provides a reasonable approximation under controlled balance conditions, where intersegmental motion is minimal. Psychological factors such as kinesiophobia (fear of movement and reinjury) were not accounted for [3], which may have compromised performance or potentially enhanced if people relied more on the automatic process. Although participants with CAI were pain‐free in the current study, we cannot fully rule out psychological effects. Detailed histories of ankle sprains and giving way could not be reported due to recall difficulties; future studies should include both retrospective and prospective tracking to test whether the identified biomechanical markers predict sprain risk. As both groups completed a similar number of balancing attempts before the first successful trial, familiarization is unlikely to account for between‐group differences; however, individual variability in familiarization with the perturbation cannot be entirely excluded. It is also possible that the nature of task failure differed qualitatively between groups, influencing the way in which participants learned from perturbations to achieve successful performance within an equivalent number of trials. Further research is needed to examine how practice influences postural control in response to repeated perturbations. Sex‐related anatomical differences can influence balance [70]. In the present study, males slightly outnumbered females, which may limit generalisability; however, potential sex‐related bias in group comparisons is considered negligible. Finally, requiring participants to keep their hands on their waists to exclude arm movements reduced individual movement variability and improved experimental control, but this also limits the applicability of findings to real‐world situations where the arms contribute to balance.

## 5 Perspective

Individuals with CAI exhibited increased MoS, characterized by delayed lateral XCoM transition and a greater XCoM‐BoS distance during platform movement compared to the HC group. This indicates that individuals with CAI exhibit less effective postural response to external perturbations. The weaker cross‐correlation between MoS and hip and trunk torque in the CAI than HC group during sudden BoS transitions suggests reduced effectiveness of dynamic temporal coupling between these segments and postural control, thereby increasing trunk effort to compensate for untimely adjustments. These findings provide a potential explanation for the kinematic alterations in proximal joints reported in the literature. Future studies should investigate the underlying neuromuscular mechanisms driving these deficits. Sudden BoS changes, such as accelerations and decelerations, are effective in identifying postural impairments in CAI. Further research is needed to determine whether these impairments can be improved through training.

## Author Contributions

Xiaohan Xu: Made contributions to the conception and design of the work, as well as to the acquisition, analysis, and interpretation of data. Also drafted the manuscript and made critical revisions to the work. Joanna Bowtell: Contributed to the conception of the work and provided significant revisions to the manuscript. William R. Young: Contributed to the conception of the work and provided significant revisions to the manuscript. Daniel T.P.Fong: Contributed to the conception of the work and provided significant revisions to the manuscript. Genevieve K.R.Williams: Made contributions to the conception and design of the work, including data acquisition, analysis, and interpretation. Also made critical revisions to the manuscript.

## Funding

This work was supported by Chinese Scholarship Council (202108310041).

## Conflicts of Interest

The authors declare no conflicts of interest.

## Supporting information


**Figure S1:** Single‐leg stance on a mediolateral moving platform.

## Data Availability

The data that support the findings of this study are available from the corresponding author upon reasonable request.
