# Supplementary figures and images for: Reduced Effective Postural Responses to External Perturbations in Chronic Ankle Instability
Source: Scand J Med Sci Sports. 2026 Jul 10;36(7):e70345. doi: 10.1111/sms.70345 (PMC13353045; doi:10.1111/sms.70345)

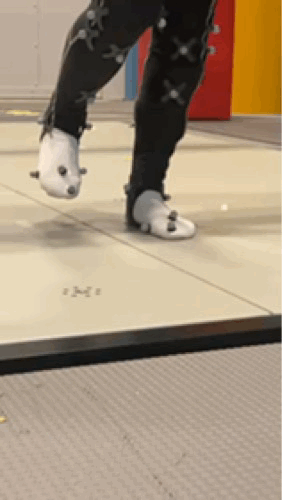


*Figure S1. Single-leg stance on a mediolateral moving platform.*

Supplement: Supplementary file 1 — Figure S1: Single‐leg stance on a mediolateral moving platform. [file SMS-36-e70345-s001.docx]
